# Healing of partial tear of the supraspinatus tendon after atelocollagen injection confirmed by MRI: A case report

**DOI:** 10.1097/MD.0000000000023498

**Published:** 2020-12-04

**Authors:** Youbin Jo, Won-Joong Kim, Heeseung Lee

**Affiliations:** Department of Anesthesiology and Pain Medicine, School of Medicine, Ewha Womans University, Ewha Womans University Mokdong Hospital, 1071 Anyangcheon-ro, Yangcheon-gu, Seoul, Republic of Korea.

**Keywords:** collagen, collagen type I, rotator cuff injury, shoulder pain

## Abstract

**Rationale:**

Recently, collagen therapy has been made available for treating rotator cuff tendon injuries. However, to our knowledge, there are no clinical studies objectively investigating the effect of collagen therapy.

**Patient concerns:**

A 53-year-old female patient visited our pain clinic because of pain in the right shoulder. Although she had never experienced trauma and had not overused her shoulder and arm, the patient showed limited range of motion with painful arc syndrome. Moreover, the Neer test and Hawkins–Kennedy test were positive with subacromial tenderness.

**Diagnoses:**

The MRI findings revealed partial tears on the articular surface of the anterior supraspinatus tendon in the rotator cuff.

**Interventions:**

The patient was treated with injections of exogenous collagen at the site of the partial tear under ultrasound guidance.

**Outcomes:**

Follow-up MRI after injection of collagen revealed healing of the previous partial rupture of the tendon without any complications. Moreover, the patient reported reduction in pain and improvement in the movement of her shoulder during the follow-up period.

**Lessons:**

In this report, we demonstrate healing of a partial tear of the supraspinatus tendon in the rotator cuff after injection of exogenous collagen, as confirmed by MRI.

## 1 Introduction

Injury of the rotator cuff tendon is one of the most common causes of pain and disability in the shoulder. There are several non-surgical treatments for this condition. Recently, type I collagen (a component of tissues, such as the skin, bone, and cartilage) has become physically available for the treatment of rotator cuff tendon injury.^[1]^ We injected exogenous type I collagen, termed atelocollagen, at the injury site for the regeneration of the tendon structure. However, currently, there are no clinical studies objectively investigating the effect of collagen therapy. To our knowledge, this is the case report confirming the recovery of the supraspinatus tendon through type I collagen therapy using magnetic resonance imaging (MRI).

## 2 Case presentation

**Figure 1:**
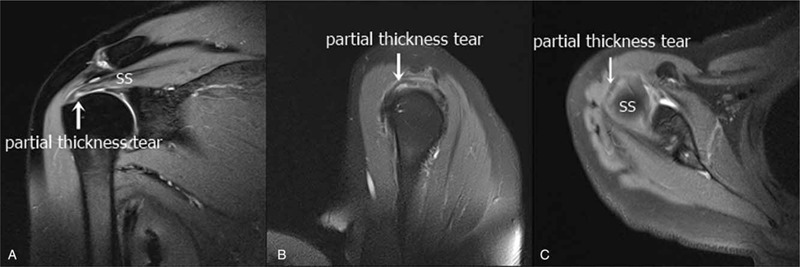
Partial tears on the articular surface of the anterior supraspinatus tendon, as shown on a shoulder MRI before the treatment. A. Coronal. B. Sagittal. C. Axial. MRI = magnetic resonance imaging, SS = supraspinatus.

A 53-year-old female (weight: 60.7 kg; height: 152 cm) visited our pain clinic with complaints of pain in the right shoulder and upper arm, lasting 1 month, with a numerical rating scale (NRS) intensity of 6. According to her medical history, she had never experienced trauma and had not performed any movements requiring overuse of her shoulder or arm. On physical examination, she had limited range of motion of the right shoulder due to the pain, which indicated painful arc syndrome. In addition, the Neer test and Hawkins–Kennedy test were positive with subacromial tenderness. Furthermore, she underwent MRI for the right shoulder prior to the initiation of treatment. The MRI result showed partial tears on the articular surface of the anterior supraspinatus tendon in the rotator cuff (Fig. 1).

Based on an initial diagnosis of a rotator cuff tear, we treated the patient with suprascapular nerve blocks and subacromial-subdeltoid bursa injections containing 10 ml of 0.75% ropivacaine hydrochloride and 40 mg of triamcinolone. We also prescribed several medications for pain control, including nonsteroidal anti-inflammatory drugs and a muscle relaxant. Within 1 week of treatment, the pain appeared to be relieved. However, she revisited our pain clinic once a month with a complaint of pain worsening (NRS 8). Repetition of the aforementioned therapy did not lead to improvement in her symptoms.

**Figure 2:**
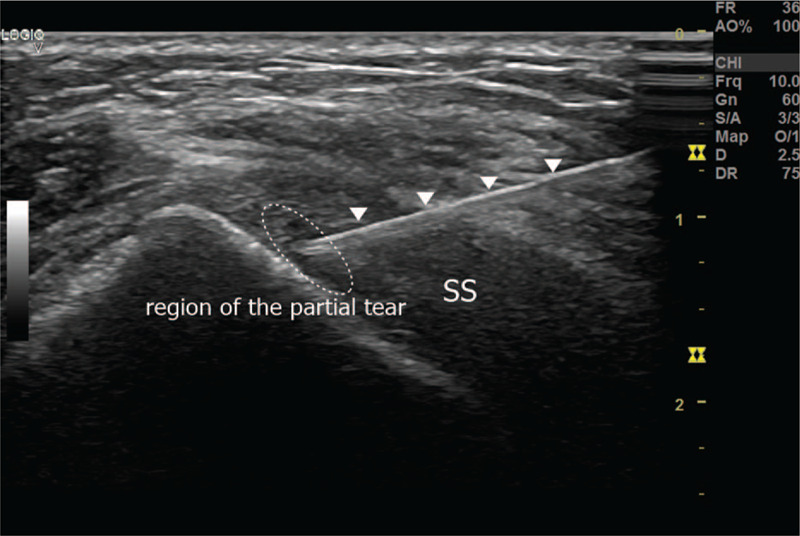
Collagen was injected in the region of the partial tear of the supraspinatus tendon where the needle reached under ultrasound guidance. A white arrowhead indicates the block needle. SS = supraspinatus.

Therefore, we decided to inject type I collagen (Coltrix Tendoregen; Ubiosis, Seongnam, Korea) into the partial tear of the supraspinatus tendon. After preparing the skin to prevent infection, we scanned the partial tear of her supraspinatus tendon under ultrasound guidance. Subsequently, we injected type I collagen (1 ml) mixed with 1 ml of 1% lidocaine into the location of the tear (Fig. 2). Type I collagen was re-injected in the same manner 1 month later.

**Figure 3:**
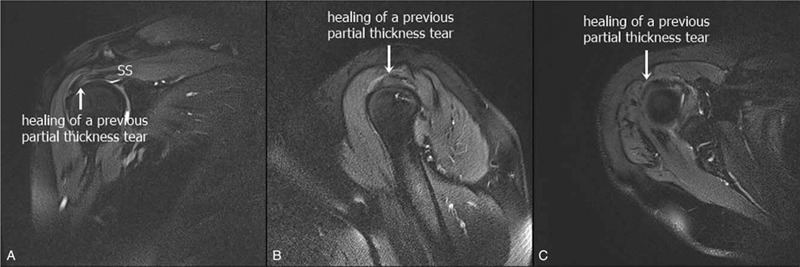
Healing of a previous partial thickness tear in the articular side of the supraspinatus tendon, as shown on a shoulder MRI after the treatment. A. Coronal. B. Sagittal. C. Axial. MRI = magnetic resonance imaging, SS = supraspinatus.

Finally, MRI of the right shoulder was performed 2 months after the first injection of type I collagen. The results confirmed the healing of the previous partial thickness tear in the articular side of the supraspinatus tendon (Fig. 3). Moreover, the patient reported a reduction in pain in the right shoulder (from NRS 8 to 2) and improvement in the movement of the shoulder. Six months later, the patient had not re-visited the hospital.

## 3 Discussion

This is the case report of treatment with exogenous type I collagen injection, for which healing of the lesion at the site of the partial tear of the supraspinatus tendon in the shoulder was confirmed by MRI.

Rotator cuff tear is one of the most common causes of pain, accounting for 40% to 60% of all cases of shoulder pain.^[1]^ Shoulder pain is exacerbated by persisting compression of the subacromial space due to weakness of structures, including the tendons and rotator cuff muscles.^[1,2]^ Occasionally, the tendons of rotator cuff muscles become irritated and inflamed, leading to compression of the subacromial bursa.^[1]^

A rotator cuff tear can be treated surgically or through conservative therapies (e.g., medication and injections of steroids). However, there are several limitations of both modalities. The administration of medicine is a simple approach to pain relief. Nonsteroidal anti-inflammatory drugs relieve pain and swelling caused by the response of the bodys immune system to inflammation in the tendons. However, the long-term use of these medications is associated with complications, including gastrointestinal problems, elevated cardiovascular risk, and liver damage.^[3]^ Conversely, it is more effective to directly inject steroids at the site of infection without other organ injury.^[3]^ Nevertheless, frequent injection of steroids also inhibits the healing process, leaving the tears of tendons at an inflamed status.^[4,5]^ In this case, we had started the treatment with the suprascapular nerve blocks followed by subacromial and subdeltoid bursa injections twice with steroid combining medications; nonetheless, the pain did not resolve. Therefore, we considered another method to treat the tear of tendon, while avoiding surgery.

Collagen is a biomaterial which is helpful in wound healing and remodeling. There are several types of collagen (types I, II, III, and V) in fibrous tissues, such as tendons, ligaments, and skin. Type I collagen, the major component of the tendon extracellular matrix (ECM) in all connective tissues, affects the structural and mechanical properties of tendon tissues.^[6,7]^ In the rotator cuff tendon ECM, type I collagen constitutes >95% of the total collagen, whereas the remaining 5% consists of collagen types III and V.^[8,9]^

Regarding the reasons for the effectiveness of collagen therapy, we expect that disorganization of the tendon ECM worsens the shoulder structure due to changes in collagen composition caused by inflammation.^[10–12]^ In addition, the ability to synthesize collagen decreases after 60 to 70 years of age, which increases the susceptibility to degenerative disease.^[1]^ Moreover, the decreasing tendency in the ratio of type I collagen to type III collagen is a major cause of ECM disorganization.^[13]^

Therefore, restoration of the tendon ECM through injection of type I collagen could heal the tear of structures. The effect is based on the induction of regenerative pathways by the synthesis of endogenous collagen and the reorganization of collagen fibers in damaged tendons.^[14]^ Injection of exogenous collagen into the lesion exerts a therapeutic effect by stimulating the restoration of fiber and induction of collagen synthesis.^[15]^ We injected an exogenous collagen, termed atelocollagen, which binds to the already existing collagen in the tissue; use of this collagen is not linked to intolerance, allergies, or infections.^[16]^ Administration of collagen at the target site activates the integrin receptors in fibroblast cell membranes. Consequently, the growth factor cascade initiates the synthesis of endogenous collagen. Finally, this pathway heals the damaged collagen fibers and leads to proper alignment.^[17–19]^

Several studies have demonstrated that type I collagen plays a major role in the proliferation of the ECM. It has been reported that type I collagen enhances the healing of rotator cuff tendon in a rabbit model using a collagen patch at the site of the tear.^[20]^ In addition, treatment with arthroscopic application of collagen for rotator cuff repair was effective.^[21]^ However, these studies were conducted using collagen patches rather than injection of exogenous collagen, which may have led to different therapeutic results.

Thus far, the effectiveness of collagen therapy was assessed by monitoring the patients symptoms after injection.^[22]^ Despite the availability of several animal and intraoperative studies on the clinical effects of collagen patches, there are insufficient investigations to objectively establish the effectiveness of exogenous collagen injection. Therefore, this case report showing tendon recovery after injection with atelocollagen, as confirmed by MRI, is clinically meaningful.

We cannot exclude the possibility that the patients partial tear of the supraspinatus tendon resolved spontaneously rather than through the injection of atelocollagen. However, there is limited knowledge regarding the natural history and spontaneous healing of partial tears. Codmans assertion^[23]^ that spontaneous healing occurs has not been substantiated by histological examination. In a series of 35 *en-bloc* histological sections from surgical specimens of partial tears, Fukuda et al did not observe active repair in any of the examined portions.^[24–27]^ Hamada et al^[28]^ reported that, similar to any other tendon of the body, there is a potential for repair of torn tendons of the rotator cuff; nevertheless, its ability for effective closure of the defect is debatable. From the clinical and histological aspects, spontaneous healing of partial tears is unlikely, except on rare occasions. Various untoward factors involved in the repair of a torn tendon include aging, separation of the tear caused by contraction and the weight of the arm, hypovascularity, shear stress within the tendon, and subacromial impingement.^[24]^

In conclusion, collagen therapy has been applied to rotator cuff tear. However, there have been no objective results regarding the use of exogenous type I collagen injection. In this case report, using MRI, we confirmed healing of the lesion at the site of partial tear of the supraspinatus tendon after injection of type I collagen.

## Author contributions

**Conceptualization:** Won-joong Kim.

**Data curation:** Won-joong Kim.

**Formal analysis:** Won-joong Kim.

**Funding acquisition:** Won-joong Kim.

**Investigation:** Won-joong Kim.

**Methodology:** Won-joong Kim.

**Project administration:** Won-joong Kim.

**Resources:** Won-joong Kim.

**Software:** Won-joong Kim.

**Supervision:** Won-joong Kim, Heeseung Lee.

**Validation:** Won-joong Kim.

**Visualization:** Won-joong Kim.

**Writing – original draft:** youbin Jo.

**Writing – review & editing:** youbin Jo, Won-joong Kim.
